# From Molecules to Behavior: Organismal-Level Regulation of Ion Channel Trafficking

**DOI:** 10.1371/journal.pbio.1000211

**Published:** 2009-09-29

**Authors:** Eric S. Fortune, Maurice J. Chacron

**Affiliations:** 1 Department of Psychological and Brain Sciences, Johns Hopkins University, Baltimore, Maryland, United States of America; 2 Department of Physiology, Center for Nonlinear Dynamics, McGill University, Montreal, Canada; 3 Department of Physics, McGill University, Montreal, Canada

Transmembrane proteins are critical, not only for cell survival, but also for a myriad of physiological functions in multicellular organisms. It is therefore necessary to have mechanisms that regulate the number of these proteins present on the cellular membrane at any given time. One such mechanism involves protein trafficking, or constitutive cycling, in which transmembrane proteins are continuously transferred between a pool located within the endoplasmic reticulum and the membrane surface by shuttle proteins (Figure 1) [1]. The actual number of proteins at the membrane surface is controlled by the rate of exocytosis (i.e., the rate at which proteins are inserted into the membrane) as well as the rate of endocytosis (i.e., the rate at which proteins are removed from the membrane). A higher rate of exocytosis will increase the number of proteins at the membrane surface, whereas a lower rate of exocytosis will decrease that number [1]–[3].

**Figure 1:**
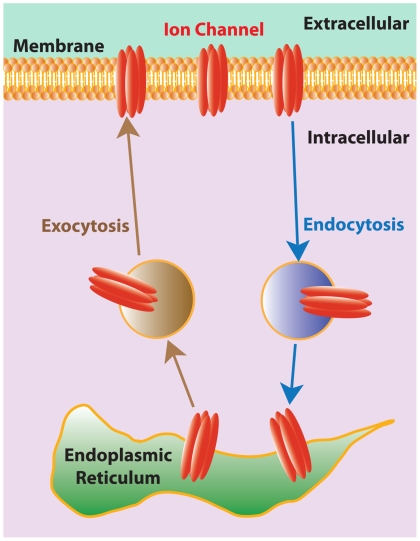
Schematic diagram of the processes involved in the trafficking of transmembrane proteins using ion channels as a example. Ion channels from a pool within the endoplasmic reticulum are moved to the membrane surface via shuttle proteins, a process called exocytosis. Ion channels can also be moved from the membrane surface back to the endoplasmic reticulum via different shuttle proteins, a process called endocytosis. Both endocytosis and exocytosis occur continuously, and one thus characterizes them by their rates, which are simply the number of transmembrane proteins being removed and inserted from the membrane per unit time, respectively. Both rates can be independently regulated.

## Receptor Trafficking as a Mechanism for the Regulation of Transmembrane Proteins

Both exocytosis and endocytosis of transmembrane proteins involve distinct agents, including chaperones, glycosylases, microtubule systems, actin, as well as myosin [1], and can be independently regulated by several known mechanisms [1]–[3], including circulating hormones [4],[5]. Ever since its proposal in the 1970s in the context of gastric acid regulation [6],[7], protein trafficking has been shown to have a crucial role in a variety of physiological functions, including the regulation of gastric acid [6],[7], osmolarity through water transport [8], glucose levels [9], and regulation of various ionic concentrations [10]–[12].

In particular, protein trafficking has also been shown to occur in excitable cells such as cardiac myocytes [2],[3] and neurons [13],[14]. The hippocampus has received perhaps the most attention in this regard; trafficking has been demonstrated to have a critical role in determining the synaptic dynamics involved in synaptic plasticity, which is thought to underlie learning and memory. Increases and decreases in AMPA receptor trafficking are correlated with long-term potentiation and depression of synapses, respectively. Further, ion channels can be inserted in and out of the neuronal membrane in a continuous fashion along with receptors during plasticity [15],[16].

As the molecular basis for channel and receptor trafficking are studied, we need to be cognizant of the cellular and organismal consequences of these mechanisms. Continuous channel and receptor trafficking appears to be a ubiquitous mechanism in both vertebrate and invertebrate animals. At first glance, this sort of trafficking of transmembrane proteins appears to be metabolically costly. What benefits, if any, do these trafficking mechanisms afford physiological systems over other mechanisms such as protein synthesis and degradation? Answering this important question will require an integrative approach that relates the molecular basis of trafficking to changes in cell function and behavior. Specialized animal model systems have historically proven to be useful in such multilevel integrative studies [17]–[19].

## Weakly Electric Fishes as a Model System for the Study of Receptor Trafficking

**Figure 2:**
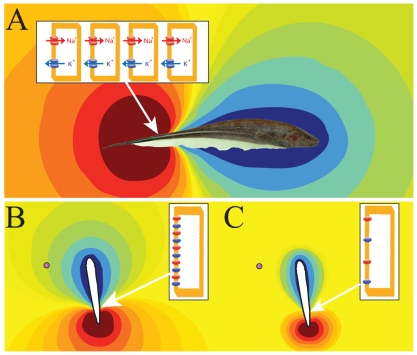
Principles of electroreception by weakly electric fish. (A) Electrogenesis in *Sternopygus macrurus*. Adult fish are on the order of tens of centimeters in length. The electric organ is located along the tail and produces a quasisinusoidal electric field whose frequency varies between 40 and 200 Hz. The electric organ, which is roughly located where the white stripe on the side of the fish appears, is composed of electrocytes with voltage-gated sodium and potassium channels that are concentrated on the caudal aspect of the cell membrane, arranged in series (inset). The currents generated by these channels summed over all electrocytes give rise to a potential difference between the inside and outside of the fish that propagates through the water at the speed of light. The colors surrounding the photograph of the fish correspond to the relative strength of the electric field. In this snapshot of the electric field, the region around the body and head is positive (blue colors) and the region around the tail is negative (red colors). At other times, the positive and negative areas are reversed. This field is detected by electroreceptors that are embedded in the skin. (B) *Sternopygus* at night or after social interactions. These conditions lead to an increase in the circulating ACTH, which increases the rate of exocytosis of channels in the electrocytes, thereby increasing their density (inset). This, in turn, increases the intensity of the electric field and, therefore, the distance at which the electric field propagates in the water. As a result, salient objects like prey items (purple dot) may be detected at greater distances. (C) *Sternopygus* during the day or in solitary conditions. Under these circumstances, there is a smaller rate of exocytosis due to lower levels of ACTH and thus fewer channels in the electrocytes (inset). As a result, the fish produces a weaker electric field that will decay over smaller distances. Because electrosensory perception is dependent on the detection of voltage differences in the water, this reduced electric field is less effective for detecting prey and for communicating to nearby conspecifics. *Sternopygus* photograph courtesy of Scott Shulz.

Weakly electric fish have a suite of simple physiological and behavioral adaptations that make them ideal for studying the physiological basis and evolution of behavior. These adaptations relate to these fishes' ability to generate an electric field (the electric organ discharge, or EOD) around their body, which is detected by electroreceptors in the skin (Figure 2) [20]. This active electric sense is used in a wide variety of evolutionary and ecologically important functions, including prey location and capture [19] and communication with conspecifics [21],[22].

The EOD results from the sum of ionic currents produced by specialized excitable cells in the electric organ called electrocytes, which are modified muscle cells [23]. In *Sternopygus macrurus*, a species of South American gymnotiform weakly electric fish commonly known as longtail or goldline knifefish, a detailed description of the ionic and molecular basis for the generation of the sinusoidal electric field has been achieved [24]. The electrocytes use a combination of specialized excitatory sodium channels and potassium channels to generate one action potential per EOD cycle: the kinetics and relative distribution of these channels are the sole determinants of EOD magnitude and duration [25],[26]. The EOD duration is used to communicate the sex and social status of an individual fish [27], whereas the EOD amplitude will effectively determine the animal's sensing volume (i.e., the volume around its body within which it can detect objects such as prey or conspecifics) as well as the emission volume of communication signals (Figure 2) [28]. Previous studies have shown that various hormones such as androgens and estrogens will modulate EOD duration through the regulation of both sodium and potassium conductances in electrocytes [29]. This level of detail between ion channel regulation in excitable cells and an easily measured behavioral output has not been described in other vertebrate systems.

## A New Functional Role for Receptor Trafficking and a New Mechanism for Its Regulation

In this issue of *PLoS Biology*, Markham et al. [30] found that social and circadian environmental factors result in dramatic changes in the amplitude of the electric field of individual *Sternopygus*: specifically, they found that *Sternopygus* increases its EOD amplitude at nighttime, when the animal is most active, hunting for prey and interacting with conspecifics.

The authors then examine the hierarchy of mechanisms that underlie this organismal-level phenomenon. Because circadian variations in the pituitary adrenocorticotropic hormone (ACTH) have been observed in a variety of vertebrate species [31], Markham et al. hypothesized that the circadian variations in the EOD amplitude of *Sternopygus* were due to changing levels of ACTH as was observed for another species of weakly electric fish, *Brachyhypopomus pinnicaudatus* [32]. The authors found that injection of exogenous ACTH into the animal leads to increases in the EOD amplitude during the day. This phenomenon can be reproduced in isolated electrocytes from these animals: the application of ACTH causes an increase in the amplitude of the action potential, which directly determines EOD amplitude.

What are the mechanisms that underlie increased action potential height? One hypothesis is that this phenomenon is due to an increased number of sodium channels at the membrane surface, which could be due to a higher rate of channel exocytosis. Markham et al. [30] show that ACTH affects a cAMP/PKA pathway to up-regulate two distinct ionic currents, a Na^+^ current and an inward rectifier K^+^ current, by increasing exocytosis of the two transmembrane molecules that mediate these currents. A delayed rectifier K^+^ current that is also found in these cells is not regulated by this mechanism. Thus, social cues lead to increased circulating ACTH, which modulates intracellular cAMP/PKA, which in turn increases the rate of sodium channel insertion into the membrane of the electrocyte. This increase in sodium channels leads to an increase in EOD amplitude, which will improve the distance at which detection of behaviorally relevant stimuli will occur [28].

## Organismal Approaches to Understanding Channel and Receptor Trafficking

The results of Markham et al. are an example of how an organismal perspective can be used to elucidate the functional roles of subcellular phenomena in evolutionarily relevant behaviors. This work has shown new modes for the regulation of ion channel trafficking, including circadian and social cues. This has important implications for the study of protein trafficking in general as environmental factors can now be used as an additional tool to study this phenomenon in other systems. Of particular interest are cardiac myocytes, which display many similarities with electrocytes [23], and sodium channel trafficking [2],[3], which can also be regulated by hormones [4].

Interestingly, Markham et al. [30] show that only two of the three ion channels present in electrocytes are up-regulated by ACTH, raising an important question regarding specificity: What makes a particular transmembrane protein a target for up-regulation or down-regulation? Furthermore, can different transmembrane proteins be trafficked by the same shuttle protein? Further studies are needed to address these issues.

The work of Markham et al. [30] also begins to address the important question of identifying the putative advantages of having constitutive cycling of transmembrane proteins, which is metabolically costly to the organism [1]. One possible advantage is that constitutive cycling permits responsiveness to circulating levels of hormones on a relatively short timescale that does not need protein synthesis [33]–[35]. We can expect that many animal systems have behaviors in which hormone titers can be expected to regulate cell excitability on a relatively fast timescale. For example, social conditions and song production are known to modulate circulating levels of hormones in songbirds [36]. These hormones, which can be regulated by the animal's behavior, in turn affect animal behavior, forming a feedback circuit from brain mechanisms through behavior. In this context, studies that examine the interplay between molecular mechanisms and behavior using the same sort of organismal approach that was used by Markham and colleagues are likely to make significant progress towards this goal.

## Abbreviations

EOD: electric organ discharge
